# Air pollution, neighbourhood and maternal-level factors modify the effect of smoking on birth weight: a multilevel analysis in British Columbia, Canada

**DOI:** 10.1186/s12889-016-3273-9

**Published:** 2016-07-16

**Authors:** Anders C. Erickson, Aleck Ostry, Hing Man Chan, Laura Arbour

**Affiliations:** 1 Division of Medical Sciences, University of Victoria, Medical Science Bld. Rm-104, University of Victoria, PO Box 1700 STN CSC, Victoria, V8W 2Y2 BC Canada; 2 Department of Geography, University of Victoria, David Turpin Bldg. Rm-B203, University of Victoria, PO Box 1700 STN CSC, Victoria, V8W 2Y2 BC Canada; 3 Center for Advanced Research in Environmental Genomics, University of Ottawa, 20 Marie-Curie, Ottawa, K1N 6 N5 ON Canada; 4 Department of Medical Genetics, University of British Columbia, Vancouver, BC Canada

**Keywords:** Maternal smoking, Multilevel models, Socioeconomic factors, Air pollution, Birth weight, Effect modification

## Abstract

**Background:**

Maternal smoking during pregnancy negatively impacts fetal growth, but the effect is not homogenous across the population. We sought to determine how the relationship between cigarette use and fetal growth is modified by the social and physical environment.

**Methods:**

Birth records with covariates were obtained from the BC Perinatal Database Registry (*N* = 232,291). Maternal smoking status was self-reported as the number of cigarettes smoked per day usually at the first prenatal care visit. Census dissemination areas (DAs) were used as neighbourhood-level units and linked to individual births using residential postal codes to assign exposure to particulate air pollution (PM_2.5_) and neighbourhood-level attributes such as socioeconomic status (SES), proportion of post-secondary education, immigrant density and living in a rural place. Random coefficient models were used with cigarettes/day modeled with a random slope to estimate its between-DA variability and test cross-level interactions with the neighbourhood-level variables on continuous birth weight.

**Results:**

A significant negative and non-linear association was found between maternal smoking and birth weight. There was significant between-DA intercept variability in birth weight as well as between-DA slope variability of maternal smoking on birth weight of which 68 and 30 % respectively was explained with the inclusion of DA-level variables and their cross-level interactions. High DA-level SES had a strong positive association with birth weight but the effect was moderated with increased cigarettes/day. Conversely, heavy smokers showed the largest increases in birth weight with rising neighbourhood education levels. Increased levels of PM_2.5_ and immigrant density were negatively associated with birth weight, but showed positive interactions with increased levels of smoking. Older maternal age and suspected drug or alcohol use both had negative interactions with increased levels of maternal smoking.

**Conclusion:**

Maternal smoking had a negative and non-linear dose-response association with birth weight which was highly variable between neighbourhoods and evidence of effect modification with neighbourhood-level factors. These results suggest that focusing exclusively on individual behaviours may have limited success in improving outcomes without addressing the contextual influences at the neighbourhood-level. Further studies are needed to corroborate our findings and to understand how neighbourhood-level attributes interact with smoking to affect birth outcomes.

## Background

Smoking during pregnancy is a modifiable risk factor associated with adverse birth outcomes and may impart long-term health consequences [1–3]. This relationship however is confounded by the presence of many other risk factors, including maternal age, education, alcohol or drug use [4–6]. Furthermore, it’s been shown that these individual-level risk factors have a dose-response association with the level of smoking, with a distinction between heavy smokers (greater than 10 cigarettes per day) and moderate or light smokers [4]. For example, while the prevalence of smoking during pregnancy decreases with increasing maternal age, the level of smoking is heavier among the older mothers who do smoke. As a result, the effect of smoking on birth weight has been shown to be modified by maternal age or other correlated risk factors [7, 8]. Similarly, neighbourhood-level factors might directly or indirectly modify the effect of smoking on birth weight such as neighbourhood deprivation or levels of particulate air pollution [9–11].

Exposure to the fine fraction of particulate matter (PM_2.5_, particles with aerodynamic diameter ≤ 2.5 μm) has shown to be a consistent risk factor associated with reduced birth weight [12]. The complex mixture of PM_2.5_ includes elemental and organic carbon compounds, metals and gases that stem predominantly from vehicle exhaust, residential heating and industrial emissions [13]. The mechanisms by which PM_2.5_ and its constituents adversely affect the reproductive system are not fully understood; however, evidence supports the potential for a shared mode of developmental toxicity with tobacco smoke exposure [14–16]. With similar chemical components, both PM_2.5_ and tobacco smoke penetrate deep into pulmonary alveolar tissues and translocate to extrapulmonary tissues causing systemic cardiovascular and immunological alterations, including platelet activation, coagulation, endothelial dysfunction, DNA damage and mutagenesis [13, 16, 17].

Low SES remains one of the most robust predictors of adverse pregnancy outcomes such as fetal growth restriction despite universal health care programs in Canada and Europe [9, 18, 19]. The society-level determinants such as poverty, poor education, income inequality and social discrimination and marginalization act indirectly on the placenta and fetus through the promotion of ‘downstream’ or mediating exposures, stresses and behaviours [20, 21]. In studies of cardiovascular disease, neighbourhood-level factors were associated with increased levels of smoking and other risk factors such as obesity, lack of exercise, lower health knowledge and lower positive behaviour changes [11, 22]. These epidemiological observations have been shown with the use of multilevel statistical models capable of separating the individual-level effects from the context of their social and physical environments [23]. The use of multilevel models in perinatal epidemiology has uncovered neighbourhood-level factors that interact with maternal-level risk factors to either buffer or mediate adverse birth outcomes [21, 24].

We present a multilevel cross-sectional analysis of birth registry data in British Columbia, Canada (population 4.6 million) to investigate neighbourhood-level differences in the effect of cigarette smoking during pregnancy on birth weight and to quantify the degree to which individual and neighbourhood-level variables explain any observed differences. Specifically, we sought to determine whether exposure to PM_2.5_ and living in low SES neighbourhoods explain between-neighbourhood differences in the effect of maternal smoking on birth weight. We also examine whether these neighbourhood-level factors modify the direct effect of maternal smoking on birth weight. Birth weight is among the most important factors affecting neonatal mortality and is a significant determinant of post-neonatal infant mortality and childhood morbidity [25]. Understanding the underlying individual and interactive effects of exposures on birth weight is crucial for effective community planning and strategic interventions to improving reproductive health outcomes.

## Methods

This was a population-based cross-sectional study of singleton births in British Columbia from 2001 to 2006 (*N* = 237,470). Data from the BC Perinatal Database Registry were provided by Perinatal Services BC (PSBC), and included information on individual-level maternal-infant health status and outcomes, reproductive history, socio-demographics, risk factors, and residential postal codes. The Registry accounts for nearly 100 % of births and stillbirths in BC of at least 20 weeks gestation or at least 500 g birth weight. Research data access is provided by a Partnership Accord /Memorandum of Agreement between all BC Health Authorities and PSBC through the *Freedom of Information and Privacy Protection Act* [26]. Research ethics board approval was granted by the University of Victoria (protocol #11-043).

The outcome variable was continuous birth weight of singleton births. Included were all births (stillbirth and live) for gestational ages of 20 to 42 weeks. Excluded birth records included: out-of-province and invalid postal codes (*n* = 1096), non-viable births prior to 20 weeks gestation or less than 500 g (*n* = 15), and the list-wise deletion of births missing important data including: cigarettes smoked per day (cigarettes/day, *n* = 2510), PM_2.5_ (*n* = 1512), birth weight (*n* = 46). Table 1 provides the full list of covariates used along with their summary statistics. All continuous independent variables, except cigarettes/day, were grand-mean centred and standardized to ease interpretation and aid model convergence. Thus, a value of zero represents the transformed variable’s mean and reference value and has a standard deviation equal to one. The variable cigarettes/day was kept un-transformed since the value zero (i.e. non-smokers) was the desired reference level. Smoking levels were capped at 20 cigarettes/day with higher values assigned a value of 20 to stabilize the distribution tail (*n* = 245, min.21 max.80). Two variables indicating the use of alcohol or drugs (prescription, non-prescription, illicit) to be a risk factor in pregnancy as identified by a physician were combined into a single dichotomous variable.

**Table 1:** Descriptive statistics^#^ for individual (Level-1) and DA (Level-2) covariates on term birth weight

Variable	Mean (sd)	Min-max
Level-1 (individual)		
Maternal age	29.8 (5.60)	11 – 55
Nulliparous	0.45 (0.50)	0 – 1
Drug/Alcohol flag	0.02 (0.15)	0 – 1
Cigarettes/day	0.79 (2.91)	0 – 20
Fall/Winter season	0.48 (0.50)	0 – 1
Level-2 (DA) Variables		
SESi	-0.08 (0.58)	-2.22 – 1.18
Education	0.50 (0.12)	0 – 0.95
Immigrant density	0.16 (0.19)	0 – 0.86
PM_2.5_	7.30 (0.86)	4.41 – 10.23
Rural address	0.11 (0.32)	0 – 1

# values shown are unstandardized, non-centered; Nulliparous: patient has never delivered a baby of at least 500 g birth weight or at least 20 weeks gestation in a previous pregnancy; Drug or Alcohol Flag: physician indicated use of drugs (prescription, non-prescription, illicit) or alcohol as risk factor in pregnancy; Cigarettes/day: number of cigarettes smoked daily at 1^st^ prenatal visit (self-reported); Fall/Winter Season: month or birth between September to February; SESi: socioeconomic status index; Education: proportion of population over 15 with any post-secondary education (trade, college, university); Immigrant Density: proportion of the population identified as immigrant status from continental Asia; PM_2.5_: Particulate Matter less than 2.5 μm; Rural: those having a rural residential address

Birth records were geocoded based on the latitude-longitude coordinate of the mother’s residential postal code at the time of delivery using *GeoRef* by DMTI [27]. Birth records were then linked to their corresponding census dissemination area (DA) by performing a point-in-polygon spatial join procedure in *ArcGIS 10.2* [28]. DAs represent the smallest geographical unit for which census data are available with a spatial coverage ranging between 200 and 800 people depending on the level of urban development. While DAs do not necessarily represent existing neighbourhood communities [29], they can act as proxies for a general catchment area of personal home-life activities [21, 30]. Birth records were identified as being either rural or urban using the Statistics Canada Metropolitan Influence Zone (MIZ) codes which are based on commuting flows of small towns into larger cities and metropolitan areas [31].

PM_2.5_ exposure was estimated using a national land-use regression (LUR) model developed to estimate PM_2.5_ at the census street block-face level [32]. The model used a number of predictors including satellite measures, proximity to major roads and industry to account for 46 % of the variability in measured annual PM_2.5_ concentrations. Individual birth records were related to the block-face point estimates using a *nearest-point* procedure in *ArcGIS10.2*. Street block-face point estimates were related to individual birth records using a *nearest-point* procedure in *ArcGIS10.2* and then aggregated to their DA-level mean to represent an area-level air pollution variable on individual births.

Three related but independent datasets all based on the 2006 Statistics Canada national census were used to represent the DA-level SES and demographic data. The first was a Canadian SES index (SESi) developed by Chan et al. which provides a measure of overall socioeconomic neighbourhood well-being [33]. The second was the proportion of population over 15 with any post-secondary education, including college, trades, or university representing higher DA-level education attainment levels. The third was the proportion of continental Asian immigrants by DA. It’s been shown in BC and elsewhere that healthy babies from Asian and South Asian backgrounds are constitutionally smaller compared to Caucasian babies [34, 35]. Asian and South Asian ethnicities are well-represented throughout BC but particularly in concentrated pockets throughout the major urban center of Metro Vancouver where levels of PM_2.5_ are also high and could therefore confound any PM_2.5_ effect. Furthermore, concentrated ethnic communities may impart buffering mechanisms through enhanced social interactions and support networks [21, 24]. A sequential regression technique was used to remove the collinearity between sets of DA-level variables [36]. Here, immigrant density was regressed against SESi and PM_2.5_ with the saved residuals representing the uncorrelated and independent contribution of immigrant density on birth weight freed from its collinearity with SESi and PM_2.5_ (r = −0.62 and 0.53 respectively). This method was repeated for SESi and education (r = 0.26) creating a residual immigrant density and residual education variable. The education and immigrant data were obtained by access to ABACUS via the Data Liberation Initiative [37].

Imputation for missing SES, education and immigrant density values was performed in order to avoid data loss of rural DAs with low population counts. Taking advantage of the nested hierarchical structure of the administrative census and health boundaries, the mean SESi value for a larger encompassing census subdivision (CSD) or local health area (LHA) was imputed for a nested DA with a missing value. There were 1441 values imputed in 52 DAs for SESi (0.6 % of final N, 0.8 % of DAs), and 3170 values imputed in 108 DAs for both education and immigrant density (1.4 % of final N, 1.7 % of DAs). Sensitivity analyses were performed using only the non-imputed data.

Hierarchical (multilevel) linear regression models were used to test our research questions, thereby accounting for the clustering, or non-independence, of individuals (level-1) belonging to a given DA neighbourhood (level-2). The multilevel model allows the intercept and slope to act as random parameters having between-area (DA) variability from an overall (BC-wide) mean intercept and slope. Therefore each DA has its own intercept and slope in which their variability from the overall mean intercept and slope can be investigated with the addition of individual (level-1) and DA-level (level-2) variables and their interactions [38]. We followed a bottom-up approach to model building to quantify the explained proportional change in variance (PCV) with the addition of sets of variables, the multilevel model equivalent to an *R* ^*2*^ [23]. We started with the empty (Null) random intercept model without any independent variables in which birth weight is only a function of the mother’s residential DA. The presence of significant random intercept variance indicates there are unexplained differences between neighbourhood means of birth weight. The proportion of the total variance in birth weight that arises due to neighbourhood differences can be quantified by computing the intra-class correlation (ICC) which represents the degree of clustering of individual birth weight within neighbourhoods [23].

The Null model was followed by Model that included the individual-level covariates as well as the addition of a random slope for the continuous variable of maternal smoking (cigarettes/day, self-reported at the first prenatal visit). By allowing cigarettes/day to be random, the mean within-DA effect of maternal smoking is allowed to differ between DAs. The presence of a significant random slope indicates that its effect on birth weight is not constant (or equal) for all DAs; that is, there are important unexplained differences between the within-DA group effects of maternal smoking on birth weight. Subsequent models included the DA-level variables along with cross-level interactions to assess their fixed effects on birth weight but to also determine if their inclusion addresses any unexplained slope variance. Several models were tested using the Akaike Information Criterion (AIC) to evaluate model performance. We report the results of three models to compare the degree of change between the level-1 and level-2 homogeneous (non-interaction) models and a model with effect-measure variation. All statistical analyses were conducted in *Stata 13IC* [39].

## Results

**Fig. 1:**
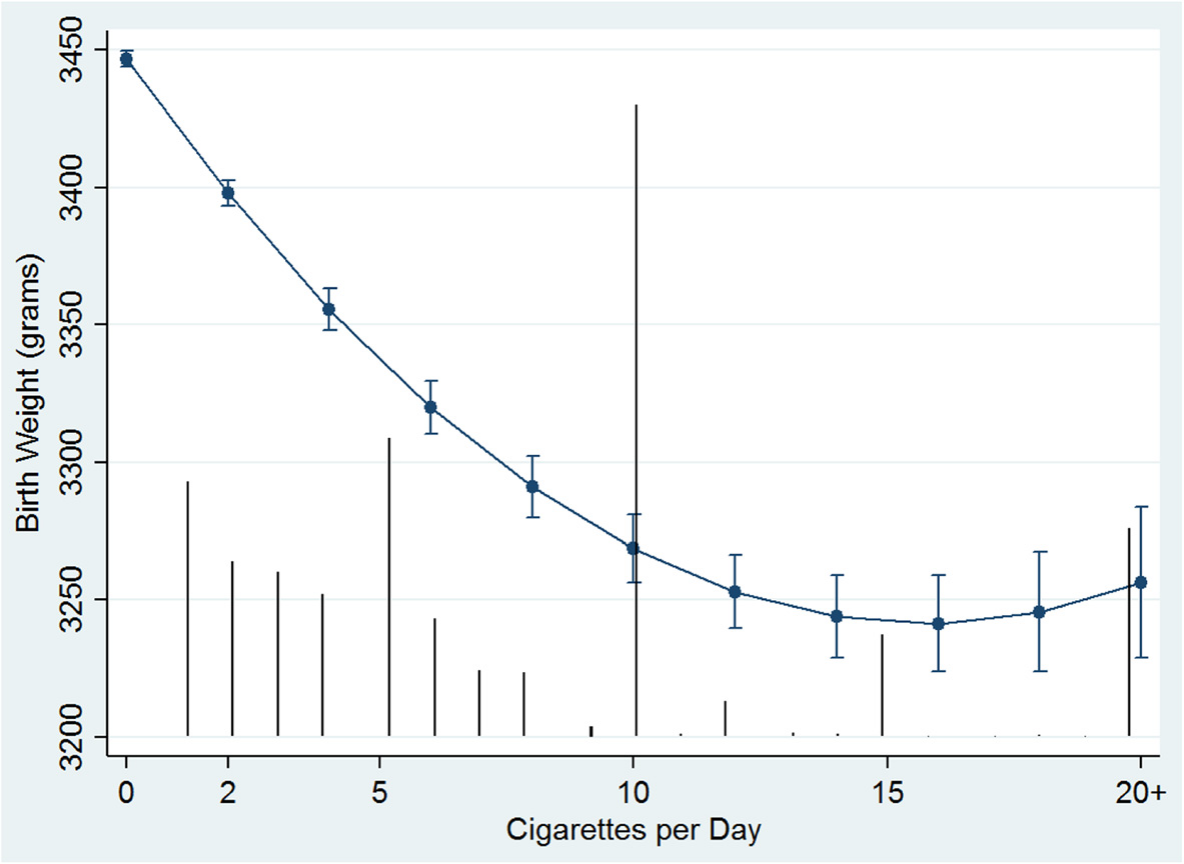
Adjusted Predicted Effects of Maternal Smoking on Birth Weight. Predicted effects of maternal smoking (cigarettes/day) on birth weight with 95 % confidence intervals are conditional on model covariates included in Model 3. *Black vertical lines* represent the frequency distribution of cigarettes/day (non-smokers, 0 cigarettes/day, have been omitted for display purposes)

After exclusions, the final dataset included 232,291 singleton (live and stillborn) births located in 6338 neighbourhood DAs (min. = 1, max. = 782, avg. = 37). Table 1 summarizes the untransformed individual and neighbourhood covariates (non-centered, non-standardized). The prevalence of maternal smoking in this population was 10.3 % (*n* = 23,836) with an average of 7.5 cigarettes/day among smokers. Table 2 reports the adjusted coefficients for the individual and DA-level covariate fixed effects on continuous birth weight (Model 1 and 2). Model 1 was a level-1 model that included only the maternal-level covariates. The relationship between birth weight and cigarettes/day was found to be non-linear and was best modeled using a quadratic term indicating a subdued dose-response with increasing exposure (Fig. 1). Model 2 added the DA-level variables. Their fixed effects show that DAs with higher SES and higher proportion of post-secondary education were significantly associated with higher birth weights; whereas DAs with increased levels of PM_2.5_, higher Asian immigrant density and rural DAs were all significantly associated with lower birth weights. Season of birth (fall or winter) was also significantly association with reduced birth weight. The results in Table 2 represent the fixed effects from homogeneous models (i.e. those without any modeled heterogeneity of the effect measure for maternal smoking).

**Table 2:** Adjusted fixed effects for level-1 and level-2 covariates on continuous term birth weight

Variables	Model 1 β (95 % CI)	Model 2 β (95 % CI)
Maternal age	-16.9 (-19.3 – -14.4)	-14.9 (-17.4 – -12.4)
Nulliparous	-107.7 (-112.5 – -103.0)	-105.5 (-110.3 – -100.7)
Drug/Alcohol flag	-171.6 (-186.9 – -156.3)	-172.2 (-187.5 – -157.0)
Cigarettes/day	-23.5 (-25.8 – -21.2)	-26.2 (-28.5 – -23.9)
cigarettes/day^a^	0.66 (0.51 – 0.80)	0.75 (0.61 – 0.90)
Fall/Winter season	-9.6 (-14.1 – -5.0)	-8.8 (-13.3 – -4.3)
SESi	–	42.7 (39.8 – 45.6)
Education	–	6.3 (3.5 – 9.1)
Immigrant density	–	-35.8 (-38.5 – -33.2)
Rural address	–	-18.8 (-28.4 – -9.2)
PM_2.5_	–	-25.0 (-28.2 – -21.8)
PM_2.5_^a^	–	3.3 (1.5 – 5.2)

See Table 1 caption for variable definitions

^a^Modeled as a quadratic

**Fig. 2:**
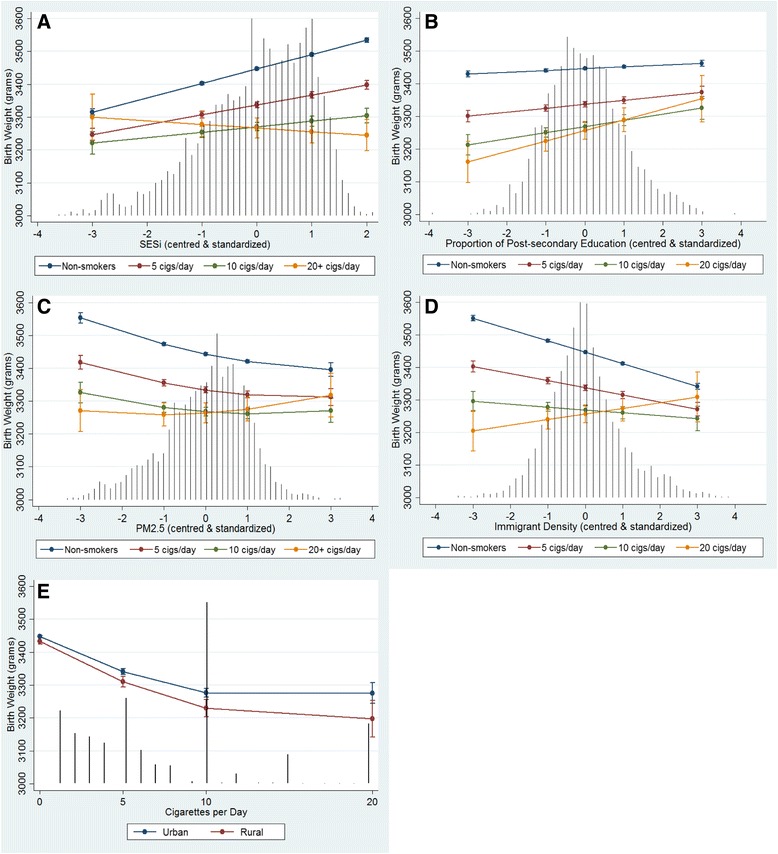
Adjusted Predicted Effects of Maternal Smoking on Birth Weight across DA-level Factors. **a** Socioeconomic Status Index (SESi) **b** Proportion of Population with Post-secondary Education **c** Particulate Matter ≤ 2.5 μm (PM_2.5_) **d** Asian Immigrant Density **e** Rural Residence. Predicted effects on birth weight with 95 % confidence intervals are conditional on model covariates included in Model 3. *Black vertical lines* represent the frequency distribution of the variable on the x-axis (except Fig. 2e which shows the frequency distribution of cigarettes/day)

Model 3 tested interactions with cigarettes/day including cross-level (level-1 by level2) and level-1 by level-1 interactions to explain the between-DA random intercept and random slope variability. The model results are presented in Table 3 including the main effects as well as the interaction effects with cigarettes/day. The degree of heterogeneity across levels of maternal smoking modified by the DA-level contextual factors is graphically presented in Fig. 2. The five graphs show the predicted conditional fixed effects of SESi, education, PM_2.5_, Asian immigrant density and rural residence on birth weight and their interactions with specified levels of maternal smoking (Fig. 2a–e respectively). For example, Table 3 and Fig. 2a show that higher SES has a significant positive association with birth weight but is less pronounced with increased levels of maternal smoking whereby very heavy smokers (≥20 cigarettes/day) do not incur any benefit of higher SES. Conversely, very heavy smokers showed the greatest gains in birth weight in DAs with higher proportions of post-secondary educated people (Fig. 2b). Recall that the higher education variable was an uncorrelated residual variable independent of SESi, and therefore these observed associations are in addition to the education-related effect captured by SESi.

**Table 3:** Adjusted individual and DA-level fixed effects on continuous birth weight and their modification by maternal smoking (Model 3)

Variables	Main effect β (95 % CI)	Modification by cigs/day β (95 % CI)	Corresponding figure
Cigarettes/day^a^	-25.7 (-28.1 – -23.3)	0.83 (0.68 – 0.98)	1
SESi	43.8 (40.9 – 46.8)	-2.7 (-3.7 – -1.6)	2A
Education	5.2 (2.3 – 8.1	1.3 (0.3 – 2.3)	2B
PM_2.5_	-26.3 (-29.6 – -23.0)	1.8 (0.9 – 2.7)	2C
PM_2.5_^a^	3.4 (1.5 – 5.3)	–	–
Immigrant density	-36.5 (-39.2 – -33.7)	2.6 (1.5 – 3.7)	2D
Rural address	-15.0 (-25.1 – -5.0)	-2.9 (-5.6 – -0.2)	2E
Maternal age	-12.1 (-14.8 – -9.5)	-2.9 (-3.6 – -2.1)	3A
Drug/Alcohol flag	-161.2 (-180.4 – -142.1)	-3.7 (-6.3 – -1.2)	3B

See Table 1 caption for variable definitions

^a^Modeled as a quadratic, value for Cigarettes/day listed under ‘Modification by cigs/day’; Model 3 covariates not listed above include: nulliparous and season of birth

Increasing PM_2.5_ levels had a significant non-linear association with reduced birth weight; however, it showed a positive interaction with maternal smoking such that the effect of increased smoking on birth weight was attenuated in DAs with higher levels of PM_2.5_ (Fig. 2c). Similarly, higher Asian immigrant density was significantly associated with lower birth weights but had a positive interaction with increased cigarette use demonstrating a protective effect of higher immigrant density DAs (Fig. 2d). Rural DAs had a significant negative interaction with maternal smoking indicating a further reduction in birth weight with increased cigarette use among rural residents (Fig. 2e).

**Fig. 3:**
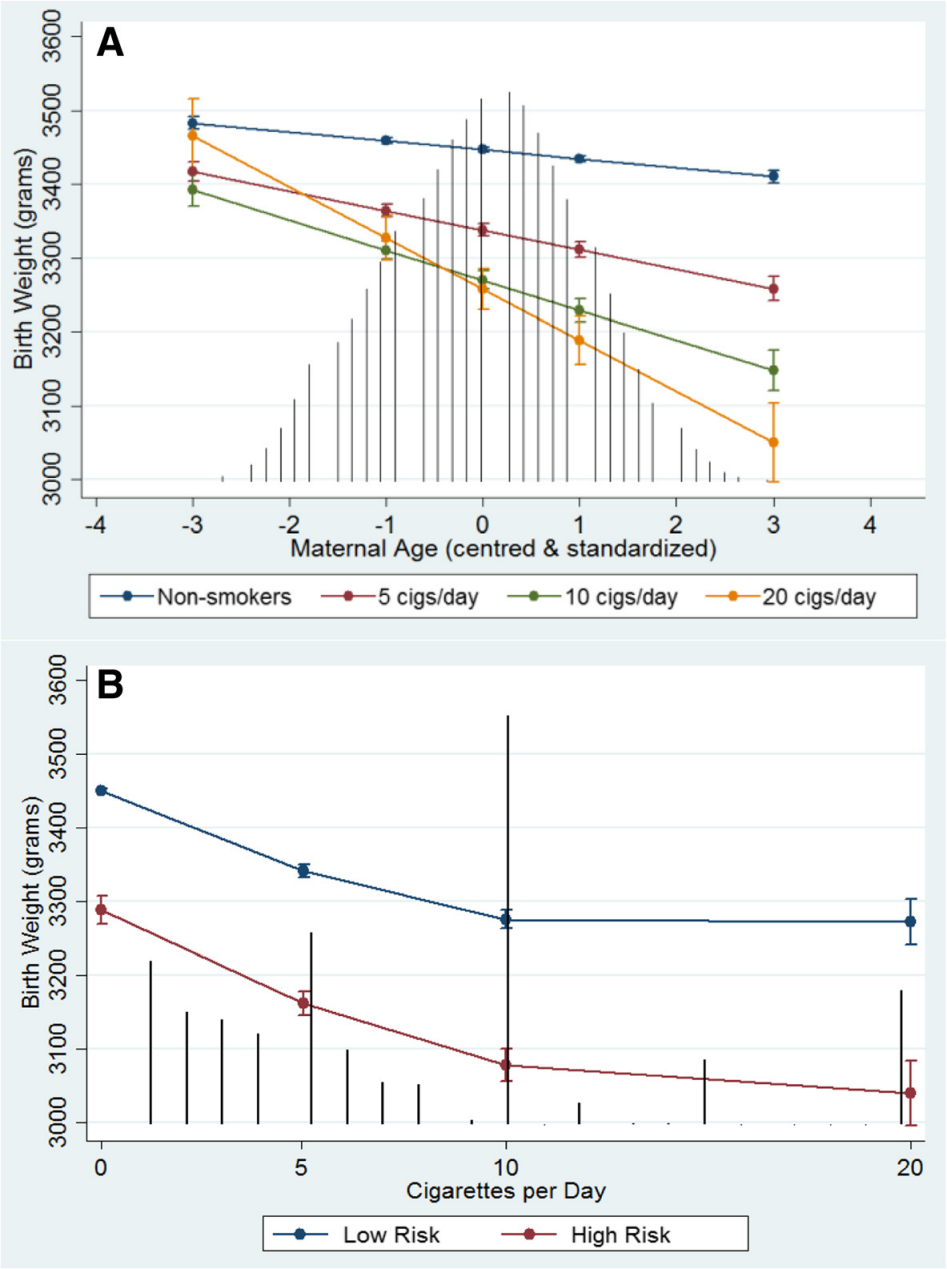
Adjusted Predicted Effects of Maternal Smoking on Birth Weight across Maternal-level Factors. **a** Maternal age **b** Suspected Drug or Alcohol Use. Predicted effects on birth weight with 95 % confidence intervals are conditional on model covariates included in Model 3. *Black vertical lines* represent the frequency distribution of the variable on the x-axis (except Fig. 3b which shows the frequency distribution of cigarettes/day)

Two level-1 interactions with maternal smoking were significant, maternal age and suspected drug or alcohol use. The predicted conditional marginal effects of these two interactions are show in Fig. 3a and 3b respectively indicating that the reduction of birth weight among heavier smokers is exasperated by older maternal age and those suspected of drug or alcohol use. A variable for neighbourhood-level smoking (DA-average cigarettes/day) was created and tested in models along with a cross-level interaction with maternal-level cigarettes/day but neither parameters were significant nor explained any additional variability.

The random effects, the explained proportional change in variance (PVC), and model diagnostics are presented in Table 4. The unadjusted ICC for the Null random-intercept model was 0.019, indicating that 1.9 % of the total residual differences in birth weight are attributable to DA-level contextual factors. However, the ICC increased to 2.2 % for Model 1 with the inclusion of the level-1 covariates and random slope for cigarettes/day. This was due to the reduction in the level-1 residual variance (560.5 to 554.7) relative to the increase in the level-2 random intercept variance (78.7 to 83.4). The (now adjusted) ICC_adj_ is conditional for the individual composition of the DAs including the random slope for cigarettes/day held constant at 0 (i.e. non-smokers). The addition of DA-level variables in Model 2 removed a lot of the DA-level variance reducing the ICC_adj_ to 0.6 %.

**Table 4:** Random Effects and Model Diagnostics

	Null model	Model 1	Model 2	Model 3
L1 residual (sd)	560.5	554.7	555.0	554.9
L2 intercept (sd)	78.7	83.4	44.4	44.2
L2 slope (sd)	–	10.7	9.8	9.0
Intercept	3434.3	3505.9	3501.8	3500.9
AIC	602672	598513	596639	596514
L1-PCV	Ref.	2.0 %	2.0 %	2.0 %
L2-PCV	Ref.	-12.3 %	68.2 %	68.5 %
ICC/VPC^#^	0.019	0.022	0.006	0.006
Int-slope corr.	–	-0.53	-0.28	-0.28

*Abbreviations*: *L1 residual (sd)* Level-1 residual standard deviation, *L2 intercept (sd)* Level-2 random intercept standard deviation, *L2 slope (sd)* Level-2 random slope standard deviation, *PCV* proportional change in variance, ^#^ *VPC* (variance partition coefficient) is equivalent to the ICC but conditional on the random-slope variable, thus values in table represent intercepts for non-smoking individuals, *Int-slope corr* intercept-slope correlation

**Fig. 4:**
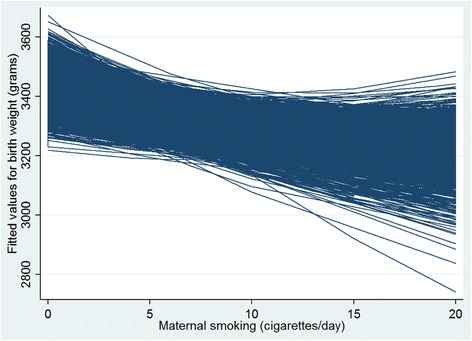
Neighbourhood-specific slopes of maternal smoking on birth weight. Empirical Bayes predictions of DA-specific regression lines for Model 1

The level-2 random intercept variance term (reported as standard deviations in Table 4) indicates that the mean birth weight for every DA has a degree of variability from the overall (BC-wide) mean birth weight. For the Null model, the overall birth weight intercept is 3434.3 g with a standard deviation of 78.7 giving an 8.6 % difference in range between 95 % of the DAs (3434.3 ± (1.96 × 78.7) = 3280.0 and 3588.6 g). The quadratic form of the random slope for cigarettes/day in Model 1 prevents a similar calculation to be performed, but Fig. 4 gives an indication of the large between-DA slope variability which shows the DA-specific slopes of maternal smoking on birth weight. The intercept-slope correlation shown in Table 4 indicates the presence of DA-level heterogeneity signifying that DAs with higher average birth weights from non-smoking mothers have a lower within-DA effect of smoking (i.e. higher average DA intercepts tend to have lower average slopes for smoking) [23, 38].

The level-1 and level-2 explained PCV (L1-PCV & L2-PCV) summarizes the relative degree of explained variance at the different levels between the different models (Table 4). Using the Null model as the reference, the Model 1 resulted in an L1-PCV of 2.0 %, and the L2-PCV in the random intercept was−12.3 %. The negative L2-PVC is a result of the larger level-2 intercept variance relative to the Null model. The addition of the DA-level variables in Model 2 explained 68.2 % of the DA-level variance compared to the Null model. Model 3 accounted for an additional 0.3 % of the L2-PCV.

Sensitivity analyses using only the non-imputed DAs (N_1_ = 229,067 in 6230 DAs) showed very minor differences in magnitude of significant variables. Most of the DAs that were missing data were in rural areas with small population numbers, the likely reason why their data were suppressed from the census tables. While some parameters were slightly attenuated, many of the interaction terms increased in magnitude. The random-intercept standard deviation was also slightly smaller than that of the same model using the full dataset while random-slope standard deviation showed no difference. In a second sensitivity analysis, we restricted the sample to only term births excluding stillbirths and congenital anomalies. As expected, there was a large reduction in the random slope variability (L2 slope (sd) = 5.7) and a small decrease in the random intercept variability (L2 intercept (sd) = 43.0) due to using only term births. Changes in the coefficients for the DA-level variables as well as their cross-level interactions with cigarettes/day were minor, within their 95 % confidence intervals reported in Table 3. The exception was the main effect of education which was no longer significant (*p* = 0.151), but its interaction with cigarettes/day did remain significant (*p* = 0.025). The maternal-level variables were attenuated but remained significant with the exception of the interaction between drug/alcohol flag and cigarettes/day which was no longer significant (*p* = 0.106).

## Discussion

This study employed multilevel random coefficient models to assess whether neighbourhood-level contextual factors can modify the effect of maternal smoking on birth weight. Our results show that the effect of maternal smoking on birth weight, self-reported as the number of cigarettes smoked per day, is modified by both individual-level and neighbourhood-level variables. However, the observed direction of the effect modification was not always as expected. Furthermore, through the use of random-slope models we show that the average effect of maternal smoking on birth weight can vary considerably between neighbourhoods which was only partially explained by the cross-level interactions. After adjusting for individual-level covariates and DA-level socio-economic, socio-demographic and air quality variables, there was a significant non-linear effect between cigarettes/day and birth weight in BC for singleton births from 2001 to 2006. This association was robust to the exclusion of stillbirths and congenital anomalies as well as the use of only term births demonstrating that selection bias does not likely affect the observed results.

The observed non-linear association between cigarettes/day and birth weight shown in Fig. 1 suggests that the largest potential effects are seen at the low to middle range of smoking levels. England et al. [40] found a very similar non-linear association of maternal smoking on term birth weight using self-reported cigarettes/day as well as using urine cotinine concentrations. Therefore, efforts to reduce the number of cigarettes smoked during pregnancy may have limited results for moderate and heavy smokers without substantial reductions or full cessation [41]. Interestingly, we found a similar curvilinear relationship with increasing levels of modeled PM_2.5_ and birth weight (Fig. 2c), a dose-response phenomenon observed in other exposure-disease contexts [42].

Beyond the non-linear association between cigarettes/day and birth weight, other factors were able to modify this relationship both positively and negatively. Our analysis confirm previously shown modification of the smoking-birth weight relationship by maternal risk factors [7, 8]; however, to our knowledge this is the first study to show that neighbourhood-level factors are able to modify this relationship. We found a significant negative interaction between cigarettes/day and neighbourhood-level SESi that resulted in the attenuation of the beneficial role of rising neighbourhood-level SES on birth weight with increased levels of maternal smoking (Fig. 2a). The predicted effects presented in Fig. 2a suggests is that maternal smoking may have little relevance in affecting birth weight in very low SES neighbourhoods, but becomes more prominent as neighbourhood-level SES increases and perhaps other stressors negatively impacting birth weight are reduced. Hence interventions focusing exclusively on individual behaviours may have limited success without addressing the contextual influences at the neighbourhood-level [9, 43–45].

Conversely, the small but significant positive interaction between higher proportions of neighbourhood-level post-secondary education and cigarettes/day found that heavy smokers may benefit the most by living in higher educated neighbourhoods (Fig. 2b). This type of cross-level effect has been observed in other epidemiological scenarios where higher risk individuals have better outcomes than would be expected due to some beneficial capacity of the neighbourhood context [11, 22]. The mechanisms by which neighbourhood-level factors affect individual health is indirectly exerted through individual-level processes, such as behaviours, adaptations and attitudes which may be transmitted between people [46, 47]. Meng et al. found that low education neighbourhoods exert an impact on low birth weight and preterm birth through unhealthy behaviours, psycho-social stress (i.e. sense of control) and SES-related support [21]. Therefore it could be that smoking cessation rates in pregnancy are higher in better educated neighbourhoods where healthier behaviours are more common [48, 49]. Fig. 2b suggests that living in higher educated neighbourhoods may encourage moderate and heavy smokers to reduce their smoking frequency to less than five cigarettes/day.

Neighbourhood social supports and transmission of behaviours could also explain the observed interactions with higher immigrant density and rural address, albeit in opposite directions. The positive interaction between higher immigrant density and maternal smoking (Fig. 2d) may reflect the buffering effect of strong community cohesiveness and beneficial cultural practices [21, 43, 47]. Conversely, the observed negative interaction between rural address and cigarettes/day (Fig. 2e) could be due to the transmission of negative behaviours due to such behaviours being more common [50], and where less support for cessation may lead to smoking throughout pregnancy [51]. The dichotomized definition used to represent rural residential addresses may obscure mechanisms which can be modified by maternal factors such as education [52].

The buffering effect of PM_2.5_ with increased levels of maternal smoking (Fig. 2c) is curious but could provide evidence for a protective pre-conditioning stress that activates an adaptive response and increases biological resistance to cigarette-induced harms [53, 54]. We found a similar positive interaction between suspected alcohol and drug use and PM_2.5_ in a different analysis [55]. The suspicion of survival bias due to competing risks was partly mitigated by using a near full population sample that included stillbirths, congenital anomalies and preterm births, although we were not able to control for fetal loss prior to 20 weeks gestation. Other explanations require further scrutiny as evidence of the opposite (negative and synergistic) effect between smoking and air pollutants has been shown [10, 16].

We have shown in an earlier paper that women who reported smoking 10 or more cigarettes/day at their first prenatal visit were significantly more likely to have other maternal risk factors, such as lower education, suspected drug or alcohol use, and fewer prenatal care visits [4]. Our current results compliment the previous study by showing that the cumulative impact of multiple risk factors can have more than an additive effect on birth weight reduction. The negative association between older maternal age and birth weight was markedly greater with increased levels of maternal smoking, particularly among the heaviest smoking group (Fig. 3a). Similarly, those who reported higher levels of smoking who were also suspected of drug or alcohol use showed a pronounced effect compared to those who reported to not smoke (Fig. 3b). These results corroborate the established literature showing similar synergistic interactions between both maternal alcohol use and smoking on lower birth weights [8, 56], as well as between maternal smoking and older maternal age on birth weight [7, 57].

While the application of multilevel models in perinatal epidemiology have become more common [58], most have been random-intercept models with very few including a random-slope parameter. Permitting the slope for the maternal cigarettes/day exposure to be random provides information on how its effect on birth weight differs between neighbourhoods and enables the search for neighbourhood-level variables to help explain the between-neighbourhood variance [38]. For example, the random-slope standard deviation presented in Table 4 drops from 10.7 in Model 1 to 9.0 in the fully adjusted Model 3. This represents a 30 % change in explained random-slope variance (10.7^2^–9.0^2^/10.7^2^). Furthermore, the addition of the level-2 variables explained 68.5 % of the random-intercept variance compared to the Null (empty) model. However in light of these findings, significant inter-DA variance remained for both the random intercept and slope.

This study used self-reports of smoking (cigarettes/day) recorded at the first prenatal visit; however, there were no data on exposure to environmental tobacco smoke or whether smoking reduction or cessation occurred during the pregnancy. The self-reporting bias of cigarette consumption can lead to the attenuation of the true effect of smoking on birth weight [59], and may therefore alter observed interactions. Studies of smoking misclassification in the United States has estimated non-disclosure to be around 20 % [60, 61]. The demographic predictors of non-disclosure include former smokers and younger maternal age which could partially explain the observed interaction between maternal age and cigarettes/day [61]. Similarly, recall bias and perceived stigma may result in under-reporting of actual consumption habits. This could account for the observed curvilinear effect on birth weight if women smoking 10 cigarettes/day report only smoking 5 per day, although England et al. observed a similar slope using urine-cotinine concentrations [40]. While relatively small, sensitivity analyses regarding the list-wise deletion of observations with missing smoking data was to exclude potentially at-risk pregnancies and could therefore alter coefficient estimates (*n* = 2501, 1.1 % of sample).

Another limitation includes potential measurement error and misclassification bias in the PM_2.5_ exposure assessment which could affect its estimates. First, the LUR PM_2.5_ concentrations may be underestimated with less variability compared to compiled monitoring data which could potentially underestimate its association with birth weight in certain areas [62]. Also the PM_2.5_ LUR model is cross-sectional based on 2006 air quality monitoring data, and we therefore assume that the study population was exposed to the same levels of PM_2.5_ across 6 year study period based on their residential DA. Finally, our analysis was based on maternal place of residence at delivery, and therefore intra-urban commuting and potential inter-urban relocation within the pregnancy period was not accounted for. Time-activity patterns show that pregnant women spend more time at home in the later stages of pregnancy, but mobility patterns may differ by age, parity and SES [63, 64].

A main strength of this study is the quality of the perinatal registry data [65]. The near 100 % ascertainment of birth records for the province of BC and quality control measures used in database management practices produces highly reliable data on maternal and newborn health outcomes, co-morbidities and exposures. However, the inability to control for individual-level SES, particularly maternal education, may influence the neighbourhood-level effect estimates and interactions. Maternal education is a variable provided in the PSBC Perinatal Registry, but was only available for 10 % of our population cohort. The adjustment for socially-patterned behavioural risk factors such as maternal smoking and suspected drug or alcohol use will control for some individual-level SES differences [4]. Notwithstanding, our results suggest that reported number of cigarettes smoked correlates with a substantial reduction in birth weight and is modified by socioeconomic, demographic and environmental risk factors suggesting the information as provided will help identify those at highest risk.

## Conclusions

The effect of maternal smoking on birth weight is not constant across geography, but rather is context specific given the social and physical environment. The use of random coefficient models revealed neighbourhood-level differences in how maternal smoking negatively impacted birth weight demonstrating effect modification by neighbourhood and maternal-level factors. The inclusion of the DA-level SES, demographic and PM_2.5_ variables explained 68.5 % of the random intercept variability in DA-mean birth weight. However, the random slope variability was only partially explained by the cross-level interactions suggesting other contextual factors are involved in determining the magnitude of maternal smoking on birth weight. Further studies are needed to corroborate our findings and to understand how neighbourhood-level attributes interact with smoking to affect birth outcomes.
